# Dry Reforming of Methane over Pyrochlore-Type La_2_Ce_2_O_7_-Supported Ni Catalyst: Effect of Particle Size of Support

**DOI:** 10.3390/molecules29081871

**Published:** 2024-04-19

**Authors:** Zeling Zhou, Chao Li, Junfeng Zhang, Qiliang Gao, Jiahao Wang, Qingde Zhang, Yizhuo Han

**Affiliations:** 1 State Key Laboratory of Coal Conversion, Institute of Coal Chemistry, Chinese Academy of Sciences, Taiyuan 030001, China; 2 University of Chinese Academy of Sciences, Beijing 100049, China

**Keywords:** pyrochlore, nickel, dry reforming, CH_4_, CO_2_

## Abstract

The properties of supports (such as oxygen vacancies, oxygen species properties, etc.) significantly impact the anti-carbon ability due to their promotional effect on the activation of CO_2_ in dry reforming of methane (DRM). Herein, pyrochlore-type La_2_Ce_2_O_7_ compounds prepared using co-precipitation (CP), glycine nitrate combustion (GNC) and sol–gel (S-G) methods, which have highly thermal stability and unique oxygen mobility, are applied as supports to prepare Ni-based catalysts for DRM. The effect of the calcining temperature (500, 600 and 700 °C) on La_2_Ce_2_O_7_(CP) has also been investigated. Based on multi-technique characterizations, it is found that the synthesis method and calcination temperature can influence the particle size of the La_2_Ce_2_O_7_ support. Changes in particle size strongly modulate the pore volume, specific surface area and numbers of surface oxygen vacancies of the La_2_Ce_2_O_7_ support. As a result, the distribution of supported Ni components is affected due to the different metal–support interaction, thereby altering the activity of the catalysts for cracking CH_4_. Moreover, the supports’ abilities to adsorb and activate CO_2_ are also adjusted accordingly, accelerating the removal of the carbon deposited on the catalysts. Finally, La_2_Ce_2_O_7_(CP 600) with an appropriate particle size exhibits the best catalytic activity and stability in DRM.

## 1. Introduction

Methane reforming with carbon dioxide, also known as dry reforming of methane (DRM), which transforms two of the main greenhouse gases (CH_4_ and CO_2_) into valuable syngas (as shown in Equation (1)), has increased hopes of efficiently utilizing natural gas and reducing CO_2_ emissions [1]. Therefore, the development of Ni-based catalysts for DRM reaction has made significant progress due to their comparable activity to noble metal catalysts.

(1) $${CH}_{4}+{CO}_{2}\to 2H_{2}+2CO,\;{\;∆H}_{298\;K}=+247\;kJ/mol$$

A prevailing consensus on the catalytic mechanism suggests that Ni-based catalysts play bi-functional roles: CH_4_ dissociation and the carbon removal with CO_2_, wherein, CH_4_ dissociation takes place on the Ni surface and carbon removal is closely related to the Lewis basic sites, and oxygen species or oxygen vacancies on the catalyst surface [2,3]. Currently, the main issue encountered regarding DRM is that catalyst deactivates gradually with time on stream, primarily attributed to Ni sintering at high temperatures and carbon deposition on the catalyst surface. To mitigate Ni sintering, some measures such as improving Ni dispersion, preparing the Ni catalyst with embedded or encapsulated configuration were often adopted [4,5,6,7,8], which has been demonstrated to be effective in suppressing Ni particle growth. The occurrence of carbon deposition is due to the unmatched rates between CH_4_ dissociation and the carbon removal, with the former exhibiting a relatively higher rate than the latter. Therefore, enhancing the abilities of CO_2_ activation and subsequent carbon removal of the employed Ni catalyst is anticipated to further balance the two rates, finally accomplishing commendable catalyst stability.

For a considerable duration, researchers have been devoting their efforts to addressing the challenge of catalyst deactivation caused by carbon deposition as well as low-temperature operation for DRM. As far as research is concerned, the anti-carbon ability of basic sites, generated through the introduction of alkali or alkaline earth metals, has been elaborated in the literature [9], and some important progress has been made. In addition, considering that oxygen species or oxygen vacancies have a positive activation effect on CO_2_, more researchers have paid attention to tuning the properties of oxygen species to investigate the origin of carbon removal. They have demonstrated that the carbonate and formate species on the catalyst surface, formed by CO_2_ adsorption and interaction with surficial oxygen vacancies and/or hydroxyl, are able to significantly contribute to the removal of intermediate carbon species. Nagaoka et al. [10] compared the reaction behaviors over Pt/Al_2_O_3_ and Pt/ZrO_2_ and observed that only a small amount of carbon deposited on the latter catalyst. They deduced that oxygen vacancies originated from ZrO_2_ due to its reducibility and then adsorbed and activated CO_2_ to form carbonate species, which finally accomplished carbon removal, wherein, following the reaction between carbonate species and intermediate carbon species, the oxygen vacancies were recovered for the next circulation. Similar to ZrO_2_, some other supports possessing oxygen storage capacity, such as CeO_2_, CeZr solid solution, etc., all have obvious promotional effects on CO_2_ activation and subsequent carbon removal. Sutthiumporn’s [11] and our results [12] demonstrated that the content of lattice oxygen in perovskite catalyst strongly affected CH_4_ and CO_2_ activation. Budiman et al. [13] found that the reorganization of lattice oxygen caused during catalyst reduction significantly improved the catalyst duration. In our previous study, we regulated the distribution of oxygen species by changing the calcination atmosphere and demonstrated that the surface-adsorbed oxygen species is beneficial for improving DRM performance due to their enhanced abilities of CO_2_ activation and carbon removal [14]. Furthermore, we doped transition metals such as Ce, La, Sm and Y into Ni/ZrO_2_ and confirmed that the surface-adsorbed oxygen species are certainly effective in promoting CH_4_ dissociation and CO_2_ activation [15]. As for the carbon removal mechanism, a widely approved viewpoint is that active oxygen species have spillover to the metal surface from the support or metal–support interface and interact with intermediate carbon species and the deposited carbon, finally completing the carbon removal. Yentenkakis et al. [3] investigated the γ-Al_2_O_3_, Al_2_O_3_-Ce_0.5_Zr_0.5_O_2-δ_ and Ce_0.5_Zr_0.5_O_2-δ_ supported Rh catalyst for DRM. They demonstrated that the unstable lattice oxygen could overflow to the surface of Rh, resulting in stabilization of the Rh^0^ and oxidization of carbon species; here, the oxygen vacancies over the support produced after oxygen spillover possess the capacity to activate CO_2_. The above convincingly demonstrated that the supports with abundant oxygen species or facile oxygen mobility are very selectable in the context of preparing the catalysts with excellent activity and stability for DRM.

Pyrochlore-type compounds, which have a typical formula of A_2_B_2_O_7_, have highly thermal stability and unique oxygen mobility. Some typical materials such as La_2_Rh_2_O_7_ have been applied for DRM reaction in some previous reports. Spivey et al. [16,17] revealed that the existing lattice oxygen in La_2_Rh_2_O_7_ support was quite conductive to carbon removal. Zhang et al. [18] reported that the high thermal stability, abundant oxygen vacancies and good oxygen mobility of A_2_B_2_O_7_ made Ni/A_2_B_2_O_7_ catalyst achieve remarkable performance.

Herein, the present study was focused on the effects of La_2_Ce_2_O_7_ supports tuned using different methods, as well as different calcination temperatures, on the catalytic performance of DRM. The origin of variations in La_2_Ce_2_O_7_ support properties and the reaction behavior caused by different preparation methods and different calcination temperatures on reaction behavior over the prepared Ni catalysts was explored in detail. The characterization results revealed that La_2_Ce_2_O_7_ prepared using a co-precipitation method with an appropriate size has more oxygen vacancies, leading to more surface-adsorbed oxygen species and higher content of basic sites for CO_2_ adsorption, which significantly contribute to better activity and stability for DRM.

## 2. Results and Discussion

### 2.1. Structure and DRM Reaction Behaviors over the Catalysts Prepared with Different Methods

#### 2.1.1. Sample Characterization

**Figure 1:**
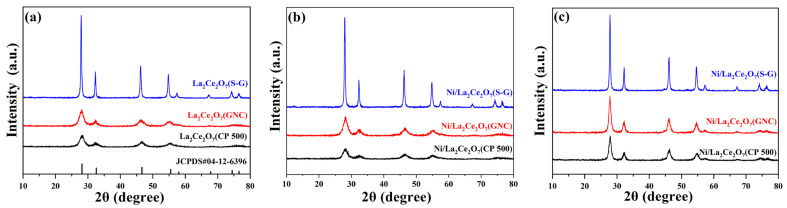
XRD patterns of the prepared La_2_Ce_2_O_7_ supports (**a**), Ni-loaded (**b**) and reduced catalysts (**c**).

Figure 1 gives XRD patterns of the prepared La_2_Ce_2_O_7_ supports and Ni-loaded and reduced catalysts. As seen in Figure 1a, all the prepared supports show characteristic diffraction peaks ascribed to pyrochlore crystalline (JCPDS#04-12-6396), without observable diffraction peaks associated with La_2_O_3_ and CeO_2_, wherein the enhanced diffraction peaks for La_2_Ce_2_O_7_(S-G) reveal that the sol–gel method is seemingly more favorable for high crystallization. This indicates that the aforementioned three preparation methods are available to prepare the pure pyrochlore crystalline. As shown in Figure 1b, the introduction of NiO has a limited effect on the pyrochlore structure, and characteristic diffraction peaks of NiO at 37.3°, 43.3°, 62.9° and 75.4° are not observable [19], indicating that the introduced NiO is highly dispersed on supports. Turning the focus to the XRD patterns of the reduced catalysts (Figure 1c), it is found that when the catalysts suffered from the reduction treatment at 700 °C, the crystalline levels of La_2_Ce_2_O_7_(GNC) and La_2_Ce_2_O_7_(CP 500) are enhanced while that of La_2_Ce_2_O_7_(S-G) has not changed, whereas there are still no diffraction peaks ascribed to metal Ni observed. Based on the XRD results, the crystalline size of the representative samples was calculated using the Scherrer equation. As listed in the right-hand column of Table 1, La_2_Ce_2_O_7_(CP 500) shows the smallest particle size (56 nm), La_2_Ce_2_O_7_(GNC) is comparable to La_2_Ce_2_O_7_(CP 500), and, nevertheless, La_2_Ce_2_O_7_(S-G) possesses a particle size as large as 229 nm. As for the reduced catalysts, the particle size of Ni/La_2_Ce_2_O_7_(CP 500) and Ni/La_2_Ce_2_O_7_(GNC) shows an obvious increase, which is attributed to the improvement of the crystalline level. This phenomenon may be because the calcination temperature is 500 °C, while the reduction temperature is 700 °C. The small particle La_2_Ce_2_O_7_ supports have undergone further sintering during the higher temperature reduction process, while the large particle support was more difficult to sinter.

**Figure 2:**
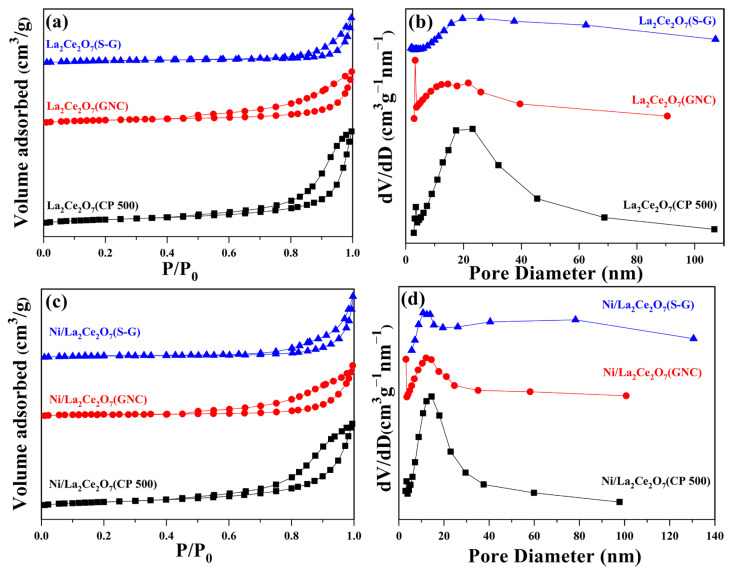
N_2_ isotherms and pore distribution of the prepared supports (**a**,**b**) and Ni-loaded catalysts (**c**,**d**).

Figure 2a–d show the N_2_ adsorption–desorption isotherms and pore size distribution of the prepared supports and Ni-loaded catalysts. From Figure 2a, it can be observed that all prepared supports show IV-type isotherms, indicating their mesoporous characteristics, whereas the relatively small H2-type hysteresis loop of La_2_Ce_2_O_7_(S-G) implies that it has a poor mesoporous structure. This phenomenon suggests that the variations in support particle size will affect the pore structure. The pore size distribution in Figure 2b further illustrates that the pore widths of La_2_Ce_2_O_7_(GNC) and La_2_Ce_2_O_7_(CP 500) are predominantly centralized within the range of 5–40 nm. In the case of Ni-loaded catalysts, it is plausible that Ni introduction rarely affects the pore mesoporous structure; nevertheless, the pore distribution is further shrunk within 5–30 nm.

Based on the N_2_ sorption data, the textual parameter of the La_2_Ce_2_O_7_ supports and supported catalysts were calculated, and the results are shown in Table 1. For the prepared support, the La_2_Ce_2_O_7_(CP 500) shows the largest BET surface area (35.2 m^2^/g), followed by La_2_Ce_2_O_7_(GNC). La_2_Ce_2_O_7_(S-G) shows the smallest BET surface area, probably attributed to its larger particle size and inadequate porous structure. The trend in change in the pore volume is consistent with BET surface, suggesting the contribution effect of the mesoporous structure. For the supported catalysts, Ni/La_2_Ce_2_O_7_(CP 500) and Ni/La_2_Ce_2_O_7_(GNC) show a decrease in specific surface area and little change in pore volume compared to their corresponding supports, while the specific surface area and pore volume have a certain growth when La_2_Ce_2_O_7_(S-G) is loaded with Ni. This indicates that the particle size of the supports affects the dispersion of the loaded Ni. Additionally, the average pore size of the catalysts after loading Ni has been improved compared to the corresponding supports, which may be due to the formation of stacked pores on the surface of Ni particles, resulting in changes in the pore distribution of the catalysts.

**Figure 3:**
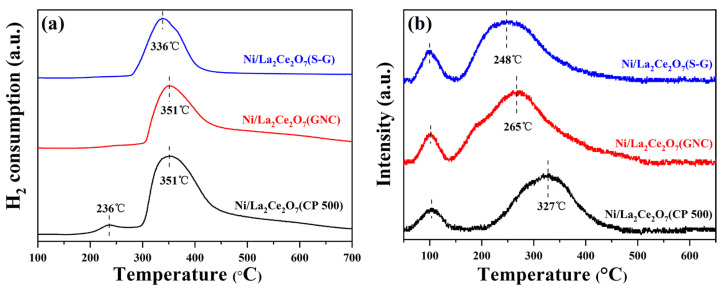
H_2_-TPR (**a**) and CO_2_-TPD (**b**) profiles of Ni/La_2_Ce_2_O_7_ catalysts.

Catalyst activity and stability are not only related to the physical properties (such as particle size, pore structure) but also affected by the interaction between active component and support [20]. The reducibility of the catalysts was characterized using the H_2_-TPR technique, and the results are shown in Figure 3a. As observed, the Ni/La_2_Ce_2_O_7_(CP 500) exhibits a major H_2_ consumption peak at 351 °C, accompanied by a satellite peak at 236 °C. Referring to the reduction peak of pure NiO, it is deduced that the small peak at low temperature is associated with the reduction of NiO particles dispersed on the support surface, which exist in an isolated state and weakly interact with the support [21], while the peak at high temperature is attributed to the reduction of the NiO interacting with the support. In theory, strong metal–support interaction is beneficial for improving Ni dispersion and enhancing the anti-sintering performance of the catalyst. The reduction peak at 351 °C on Ni/La_2_Ce_2_O_7_(GNC) is quite similar to that on Ni/La_2_Ce_2_O_7_(CP 500), whereas the peak area on the former is obviously smaller than that on the latter. This indicates that there are more reducible Ni species over the Ni/La_2_Ce_2_O_7_(CP 500). The lowest reduction temperature (336 °C) of Ni/La_2_Ce_2_O_7_(S-G) suggests the weakest interaction between NiO and the La_2_Ce_2_O_7_(S-G) support. We deduce that the particle size of supports affects the state of oxygen species on the catalyst surface and porous properties, consequently impacting the existence state of Ni species, thereby resulting in distinct reduction behaviors over the three catalysts.

The basicity properties of the catalyst prepared with different methods were investigated using the CO_2_-TPD technique. For each operation, the sample was first pre-reduced at 700 °C. Figure 3b presents the CO_2_-TPD curves. As observed, the three catalysts all exhibit two CO_2_ desorption peaks, wherein the one at 100 °C is ascribed to the weak basic sites on the catalyst surface, which could be assigned to the reactive bicarbonates originated from weak interactions between hydroxyl groups on pyrochlore surface and CO_2_ [22], and the other one is related to strong adsorption of CO_2_. Compared to the desorption at high temperature, it is apparent that Ni/La_2_Ce_2_O_7_(CP 500) possesses the strongest basicity within three catalysts, and Ni/La_2_Ce_2_O_7_(S-G) has the weakest basicity. For DRM reaction, the ability of CO_2_ adsorption strongly affects the catalytic performance. Specifically, the robust adsorption of CO_2_ is able to furnish relatively many oxygen species to remove inert carbon species at the transition state, and it can alleviate the accumulation of the carbon deposit, thus prolonging the catalyst’s lifetime [23]. Based on the above, the strong basicity on Ni/La_2_Ce_2_O_7_(CP 500) is expected to exert a positive effect on DRM running over a long period.

**Figure 4:**
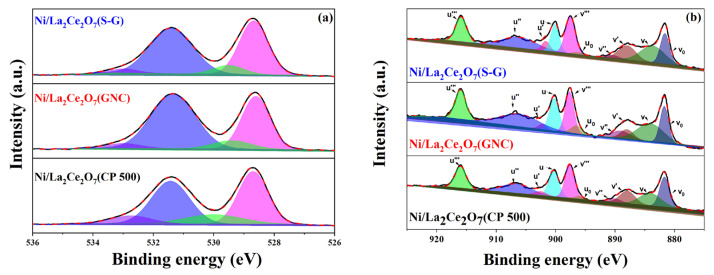
(**a**) XPS profiles of reduced Ni/La_2_Ce_2_O_7_ catalysts (O1s); (**b**) XPS analysis of Ce^3+^/Ce^4+^ on the surface of Ni/La_2_Ce_2_O_7_ catalysts.

To understand the existing state of the component in a catalyst, the reduced catalysts were characterized using the XPS technique. Figure 4a shows XPS spectra of O1s and the corresponding fitted results, where the peak at 528.6 eV is ascribed to surface lattice oxygen species [14] and the one at 531.5 eV is associated with carbonate species [24]. Moreover, another two peaks at 533 and 529.5 eV are also observed, which are thought to be related to superoxide ions (O_2_^−^) and peroxy ions (O_2_^2−^), respectively [25]. Some research has indicated that electrophilic oxygen species (O_2_^−^, O_2_^2−^) are closely related to the oxygen vacancy on the surface [26]. Here, the percentage of oxygen vacancy can be presented as (O_2_^−^ + O_2_^2−^)/O^2−^ [27,28]. Another study also indicated that oxygen vacancies are able to play remarkably positive roles in absorbing and activating CO_2_ [29,30], which is effective to suppressing carbon deposition. The percentage of different oxygen species on the surface is normalized and listed in Table 2. As observed, the value of (O_2_^−^ + O_2_^2−^)/O^2−^ declines in the following order: Ni/La_2_Ce_2_O_7_(CP 500) > Ni/La_2_Ce_2_O_7_(GNC) > Ni/La_2_Ce_2_O_7_(S-G). This suggests that the most oxygen vacancies are presented on Ni/La_2_Ce_2_O_7_(CP 500), thereby facilitating the adsorption and activation of CO_2_.

For the catalysts containing a Ce component, as reported in the literature, the amount of electrophilic oxygen species is related to the concentration of Ce^3+^ on the surface [31,32]. Figure 4b presents the Ce2p XPS spectra of the reduced catalysts, wherein the fitted peaks for v_0_, v′, u_0_ and u′ are related to Ce^3+^ species, while the peaks for v, v″, v‴, u, u″ and u‴ are associated with Ce^4+^ species. The discernible binding energies assigned to Ce^3+^ and Ce^4+^ species are evidently observed, due to the facile transformation from Ce^3+^ and Ce^4+^. Based on the obtained fitted result, we subsequently determined the percentage of Ce^3+^ using the following equation [31,32], and the corresponding results are tabulated in Table 2. It is observed that the Ce^3+^ percentage is arranged in the following order: Ni/La_2_Ce_2_O_7_(CP 500) > La_2_Ce_2_O_7_(GNC) > La_2_Ce_2_O_7_(S-G), which is consistent with the sequence of the amount of oxygen species. This indicates that oxygen species on the catalyst surface are closely related to the valence of Ce species.

(2) $${Ce}^{3+}\left(\%\right)=\frac{{Ce}^{3+}}{{Ce}^{3+}+{Ce}^{4+}}\times 100\%$$

Based on the XPS results, the Ni elemental concentration was obtained. As seen in Table 2, the most Ni content is presented on Ni/La_2_Ce_2_O_7_(CP 500), adjacent to that on Ni/La_2_Ce_2_O_7_(GNC), whereas Ni/La_2_Ce_2_O_7_(S-G) shows the minimum Ni content, probably attributed to the immersion of partial Ni into the bulk.

**Figure 5:**
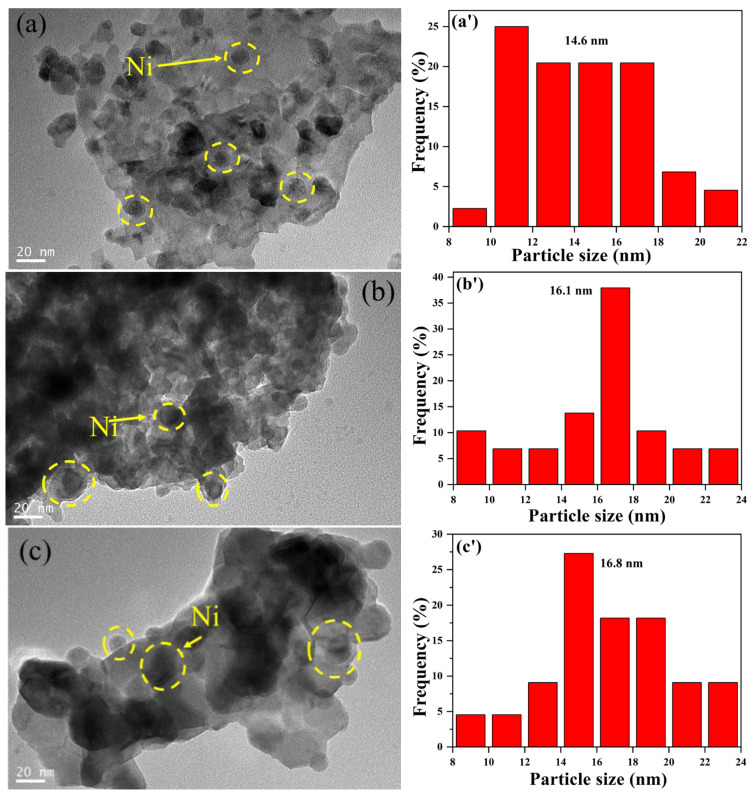
TEM images of the reduced catalysts: (**a**) Ni/La_2_Ce_2_O_7_(CP 500), (**b**) Ni/La_2_Ce_2_O_7_(GNC), (**c**) Ni/La_2_Ce_2_O_7_(S-G); particle size distribution of the reduced catalysts: (**a’**) Ni/La_2_Ce_2_O_7_(CP 500), (**b’**) Ni/La_2_Ce_2_O_7_(GNC), (**c’**) Ni/La_2_Ce_2_O_7_(S-G).

The morphology of the catalysts was observed via TEM characterization, as shown in Figure 5. For Ni/La_2_Ce_2_O_7_(CP 500) (Figure 5a), Ni nanoparticles are evenly distributed on the La_2_Ce_2_O_7_(CP 500) support, and the average size of particles is centered at 14.6 nm. In the case of Ni/La_2_Ce_2_O_7_(GNC) and Ni/La_2_Ce_2_O_7_(S-G), obvious growth of Ni particles occurred and particle sizes of Ni on them increased to 16.1 and 16.8 nm, respectively, which is attributed to their relatively large particle size for supports and small BET area. From the particle distribution plot in Figure 5c, it is further worth noting that Ni particle is not well dispersed on Ni/La_2_Ce_2_O_7_(GNC).

Based on the above, it is concluded that La_2_Ce_2_O_7_ supports prepared using different preparation methods have different particle sizes, in the order of Ni/La_2_Ce_2_O_7_(S-G) > Ni/La_2_Ce_2_O_7_(GNC) > Ni/La_2_Ce_2_O_7_(CP 500). The smaller the size of the support particles, the larger the BET surface area, the more developed the pore structure, the stronger the metal–support interaction, and the richer the surface O vacancies are. Moreover, supports below 100 nm increase in particle size after loading Ni and reducing at 700 °C.

#### 2.1.2. DRM Performance of the Ni/La_2_Ce_2_O_7_ Catalyst

**Figure 6:**
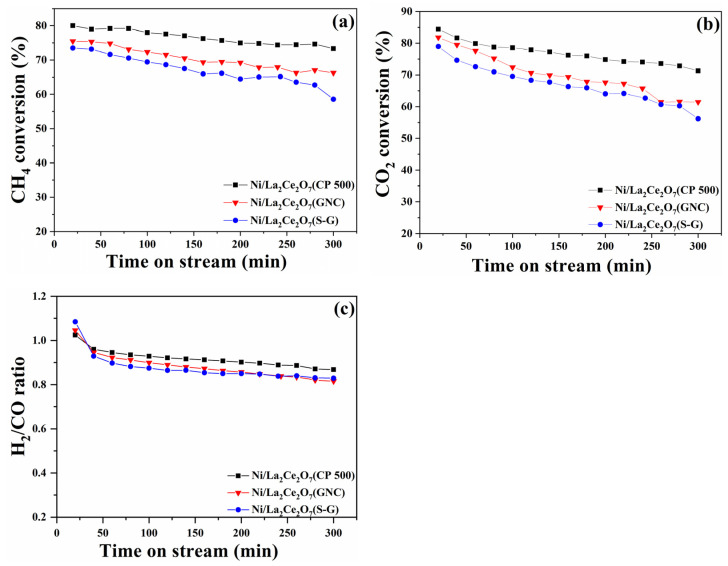
Dependence of performance of the prepared Ni/La_2_Ce_2_O_7_ on reaction time: (**a**) CH_4_ conversion, (**b**) CO_2_ conversion, (**c**) H_2_/CO ratio. (reaction conditions: 700 °C, GHSV = 48,000 mL/(g∙h) with CH_4_/CO_2_ = 1/1).

Figure 6 gives the conversions of CH_4_ and CO_2_ and the ratio of H_2_/CO as a function of time on stream. The CH_4_ and CO_2_ conversions decrease inordinately on the three catalysts with the increase in reaction time. In the evaluation period, Ni/La_2_Ce_2_O_7_(CP 500) shows the optimal catalytic performance, followed by Ni/La_2_Ce_2_O_7_(GNC) and Ni/La_2_Ce_2_O_7_(S-G). Further comparing the deactivation rate, the activity of Ni/La_2_Ce_2_O_7_(CP 500) is seemingly somewhat alleviated, suggesting that it is more durable than the other two. The above characterizations revealed that different preparation methods caused different support particle sizes, which strongly affected the catalyst performance. Since the La_2_Ce_2_O_7_(CP 500) support possesses a relatively larger BET area and a well-developed pore structure, the formed highly dispersed Ni particles on the support surface certainly favor for CH_4_ dissociation. Simultaneously, the more oxygen vacancies and strong basic sites over Ni/La_2_Ce_2_O_7_(CP 500) are conductive to the adsorption and activation of CO_2_, further promoting the removal of carbon. In Figure 6b, higher CO_2_ conversion on Ni/La_2_Ce_2_O_7_(CP 500) compared to the other two catalysts reflects its more superior promotional impact on CO_2_ reactivity. For the Ni/La_2_Ce_2_O_7_(S-G), as indicated by the above characterizations, the relatively large particle size for support results in inadequate porous structure and BET area. Consequently, there is insufficient dispersion of the introduced Ni components, together with weak interaction with active Ni and support and basic sites and low content of oxygen vacancies; as a result, the catalytic performance is the poorest. As shown in Figure 6c, the ratio of H_2_/CO is below 1 due to the RWGS reaction ($H_{2}+{CO}_{2}\to H_{2}O+CO$) and decreases with time on stream [22], wherein the ratio value of H_2_/CO represents the level of RWGS. Seemingly, a more severe RWGS reaction proceeds on Ni/La_2_Ce_2_O_7_(S-G) than the other two.

#### 2.1.3. Characterization of the Spent Catalyst

**Figure 7:**
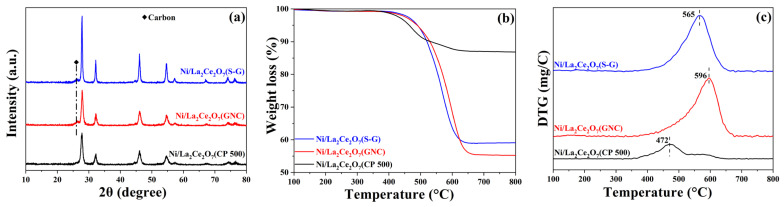
XRD profiles (**a**), TG profiles (**b**) and DTA profiles (**c**) of the spent Ni/La_2_Ce_2_O_7_ catalysts.

In order to investigate the cause of La_2_Ce_2_O_7_-supported catalyst deactivation, the spent catalysts were characterized. Figure 7a shows the XRD spectra of Ni/La_2_Ce_2_O_7_ catalysts after the reaction. As seen, the spent Ni/La_2_Ce_2_O_7_ catalysts exhibit similar diffraction peaks to the fresh ones, indicating that the original crystalline shape is well kept. For the spent Ni/La_2_Ce_2_O_7_(S-G) and Ni/La_2_Ce_2_O_7_(GNC) catalysts, the diffraction peaks of graphitic carbon appear at 26.3°, denoting that there is a more obvious occurrence of carbon accumulation on these two catalysts, whereas there is no diffraction peak attributed to graphitic carbon over the Ni/La_2_Ce_2_O_7_(CP 500) catalyst, demonstrating its good carbon deposition resistance. Figure 7b shows the thermo-gravimetric curve of the spent catalysts. It is evident that the Ni/La_2_Ce_2_O_7_(CP 500) catalyst exhibits a weight loss of only 13.17%, showing a high resistance to carbon accumulation. In contrast, the Ni/La_2_Ce_2_O_7_(S-G) and Ni/La_2_Ce_2_O_7_(GNC) catalysts manifest higher carbon accumulation, reaching approximately 45%. This also corresponds to the above XRD results.

The activity of the catalyst surface-deposited carbon was determined from the peaks in the DTA curves. As shown in Figure 7c, the peaks of the DTA curves for the three catalysts appear during two different temperature intervals. According to the literature [33,34], three types of carbon are formed after CH_4_ cracking: C_α_, C_β_ and C_γ_, and the order of oxidation activity is as follows: C_α_ > C_β_ > C_γ_. Specifically, the Ni/La_2_Ce_2_O_7_(CP 500) catalyst has a lower exothermic peak at 472 °C attributed to the more active C_β_, which can react with CO_2_ to form CO. However, it should be kept in mind that the formed C_β_ encapsulates the active Ni component, thereby causing catalyst deactivation. In the case of the Ni/La_2_Ce_2_O_7_(S-G) and Ni/La_2_Ce_2_O_7_(GNC) catalysts, exothermic peaks classified as C_γ_ in the high-temperature region were observed, which is graphitic carbon [35]. Owing to its high inertness, graphitic carbon has relatively low reactivity and is difficult to gasify with CO_2_ under reaction conditions. With a prolonged reaction time, graphite carbon is further formed and wrapped around the active Ni particles, which prevents reactant molecules from accessing the active center, ultimately accelerating the deactivation of the catalyst [36,37].

As illustrated in Figure 6, the Ni/La_2_Ce_2_O_7_(S-G) and Ni/La_2_Ce_2_O_7_(GNC) catalysts deactivate faster. It is widely recognized that the key factors leading to the deactivation of Ni-based catalysts are the sintering of Ni and carbon deposition. No obvious diffraction peaks of Ni are observed from the XRD spectra of the spent catalysts. This indicates that the sintering of Ni on this catalyst was not serious. Thus, it is plausible that carbon deposition is the main reason for the faster deactivation.

### 2.2. DRM Reaction Behaviors over Ni/La_2_Ce_2_O_7_(CP T) Catalysts Obtained through Calcination at Different Temperatures

Through the aforementioned investigation, we have realized that the Ni/La_2_Ce_2_O_7_(CP 500) catalyst exhibits the best catalytic performance in DRM due to a relatively smaller support particle size. Furthermore, pre-reduction in 700 °C may induce the aggregation of the small particles of the support. The impact of the calcination temperature on the support particle size and reactivity of the corresponding Ni-based catalyst in the DRM reaction remains unclear. Herein, in this section, the Ni/La_2_Ce_2_O_7_(CP, T) with different calculation temperatures (500, 600, and 700 °C) was utilized to further investigate the effect of support particle size on the performance in the DRM reaction.

#### 2.2.1. Sample Characterization

**Figure 8:**
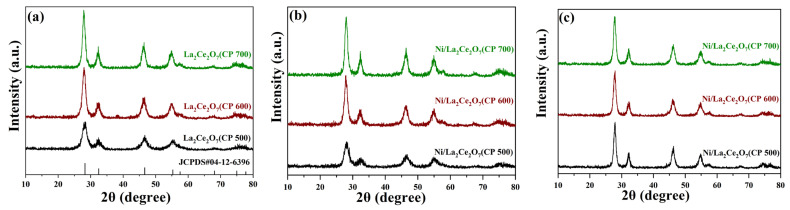
XRD patterns of the prepared La_2_Ce_2_O_7_ (CP T) supports (**a**), Ni-loaded (**b**) and reduced (**c**) catalysts.

Figure 8 displays the XRD patterns of the supports calcined at different temperatures and the Ni-loaded and reduced catalysts. From Figure 8a, the intensity of the diffraction peaks is enhanced with the elevation of the calcination temperature, suggesting that the calcination temperature led to the increase in crystal size of the supports, as also indicated in Table 3. For the supported catalysts (Figure 8b), the absence of diffraction peaks attributed to NiO indicates that NiO has been highly dispersed on the supports. Surprisingly, three catalysts suffering from H_2_ pre-reduction show a comparable intensity in terms of the diffraction peak. It is deduced that the support was aggregated into larger crystals due to pre-reduction at 700 °C. No diffraction peaks of metal Ni suggests that the reduction treatment does not affect the Ni dispersion. The above results imply that the higher the temperature is, the larger the support particle size is, regardless of the calcination or reduction process.

The samples are further characterized through N_2_ sorption at low temperature. The isotherms reveal that all samples maintained a good mesoporous structure, whereas Ni introduction slightly narrows the pore diameter distribution (Figure S1). As seen in Table 3, the BET surface area decreases with the increase in calcination temperature. The changes in BET surface area after loading Ni on the three supports are not consistent. These differences indicate that dispersion of Ni is affected by the particle size of the support. Although the pore volumes of all three supports increase after loading Ni, the changes in La_2_Ce_2_O_7_(CP 500) and La_2_Ce_2_O_7_(CP 600) are almost negligible, while the changes in La_2_Ce_2_O_7_(CP 700) are significant. Differences in pore volume changes before and after loading Ni on three pyrochlore supports also demonstrate the influence of the support particle size on the Ni distribution.

**Figure 9:**
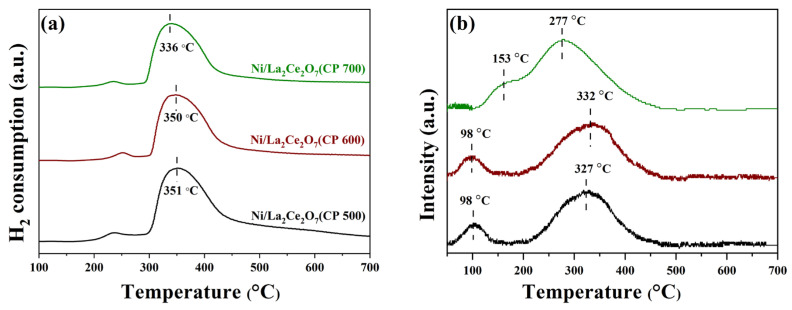
H_2_-TPR (**a**) and CO_2_-TPD (**b**) profiles of the Ni/La_2_Ce_2_O_7_ (CP T) catalysts.

H_2_-TPR results of the Ni/La_2_Ce_2_O_7_(CP T) catalysts are shown in Figure 9a. As observed, the Ni/La_2_Ce_2_O_7_(CP 500) catalyst has a higher peak hydrogen consumption temperature, followed by Ni/La_2_Ce_2_O_7_(CP 600), and Ni/La_2_Ce_2_O_7_(CP 700) has the lowest reduction temperature. However, the temperatures of the H_2_ consumption peaks of Ni/La_2_Ce_2_O_7_(CP 500) and Ni/La_2_Ce_2_O_7_(CP 600) catalysts are very close to each other, indicating that the interactions between the active component and the support on both catalysts were very close. The shift in the H_2_ consumption peak of the Ni/La_2_Ce_2_O_7_(CP 700) to a lower temperature suggests that the interaction is weak on this catalyst, ascribed to the fact that larger support particles may lead to weaker metal–support interactions.

Figure 9b shows the CO_2_-TPD results of the Ni-loaded La_2_Ce_2_O_7_(CP T) samples. Three catalysts have two CO_2_ desorption peaks; Ni/La_2_Ce_2_O_7_(CP 500) and Ni/La_2_Ce_2_O_7_(CP 600) feature a desorption peak located at 100 °C representing weakly basic sites while the desorption peak at 332 °C is associated with the strongly basic sites. Additionally, the desorption peak of Ni/La_2_Ce_2_O_7_(CP 600) representing the strongly basic sites shows an obviously larger area, demonstrating that the number of strongly basic sites on this catalyst is higher, which is beneficial for CO_2_ adsorption. For the Ni/La_2_Ce_2_O_7_(CP 700) catalyst, the desorption peaks, ascribed to moderately strong basic sites, shifts to a low temperature (277 °C), and the degree of overlap between the two desorption peaks is reinforced. This indicates a slight weakening of the basicity of Ni/La_2_Ce_2_O_7_(CP 700), which is unfavorable for CO_2_ adsorption.

**Figure 10:**
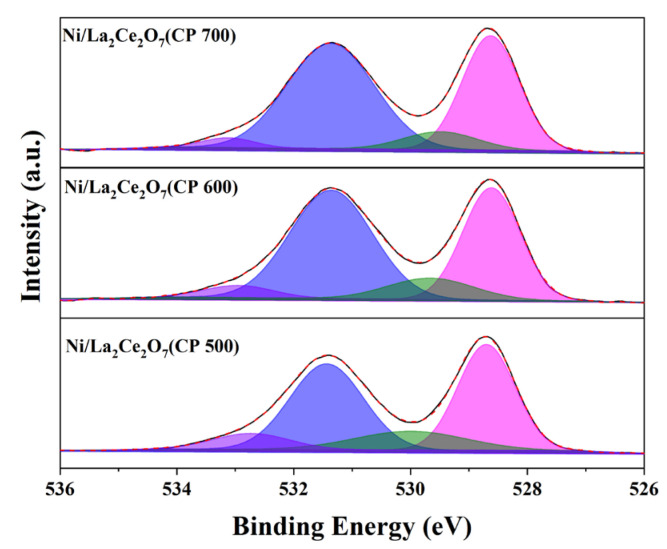
XPS profiles of reduced Ni/La_2_Ce_2_O_7_ catalysts (O1s, violet: O_2_^−^. Blue: CO_3_^2−^, olive: O_2_^2−^, magenta: O^2−^).

To further investigate the content and nature of oxygen species on the catalyst surface, XPS measurement was performed. The O1s spectra of the reduced catalysts are shown in Figure 10, and the quantities of surface oxygen species are accordingly tabulated in Table 4. According to the literature [14], XPS spectra of O1s located at low binding energies correspond to lattice oxygen species, while those at high binding energies are related to adsorbed oxygen species. As observed, the Ni/La_2_Ce_2_O_7_(CP 600) catalyst has the highest content of oxygen vacancies on the surface, followed by Ni/La_2_Ce_2_O_7_(CP 500), with Ni/La_2_Ce_2_O_7_(CP 700) showing the least amount. XRD results (Figure 8) indicated that pre-reduction made the crystal size of the support with different calcination temperatures converge to the same. Based on the XPS results, it appears that this pre-treatment also affected the re-distribution of oxygen species on the catalyst surface.

The relative content of adsorbed oxygen species plays a significant role in both the cleavage of CH_4_ and the activation of CO_2_. Generally, the cleavage of CH_4_ is mainly influenced by the size and activity of the Ni particles and the interaction of the supports [38,39,40]; however, when the difference between these two factors is not substantial, the cleavage of CH_4_ is then correlated to the adsorbed oxygen species, which is able to promote the cleavage of CH_4_ [14]. From Table 4, the Ni/La_2_Ce_2_O_7_(CP 600) catalyst after reduction exhibits the largest peak area corresponding to adsorbed oxygen species, i.e., the highest relative content of adsorbed oxygen species, while the relative content of lattice oxygen shows the following trend in the relative content of adsorbed oxygen species on the surface of the three catalysts: Ni/La_2_Ce_2_O_7_(CP 600) > Ni/La_2_Ce_2_O_7_(CP 500) > Ni/La_2_Ce_2_O_7_(CP 700). The above results indicate that the Ni/La_2_Ce_2_O_7_(CP 600) catalyst is able to better catalyze CH_4_ and CO_2_ when the effect of Ni is factored out.

**Figure 11:**
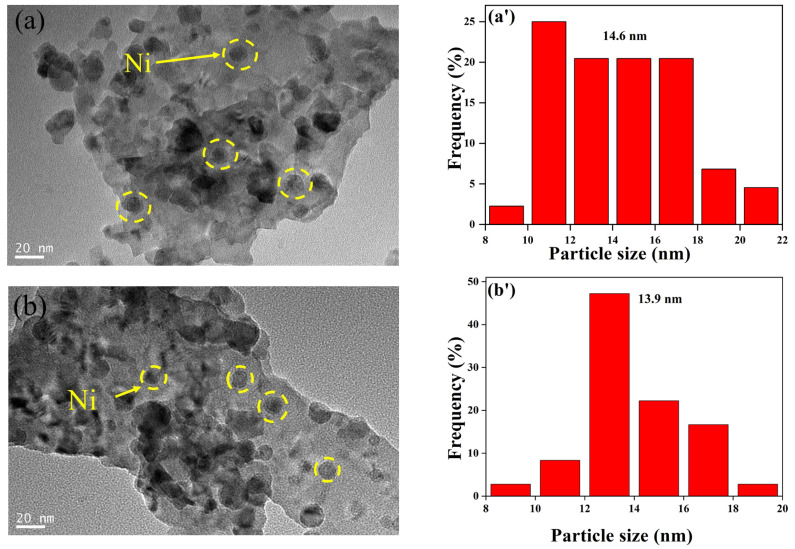

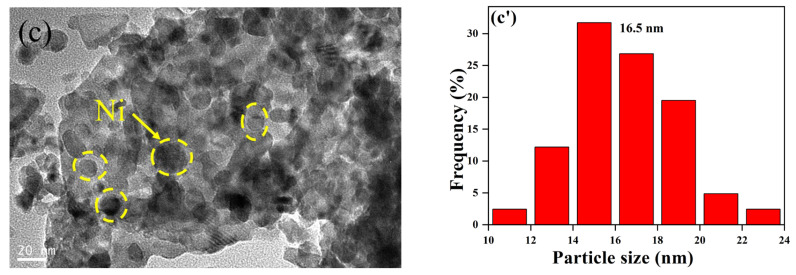
TEM images of reduced (**a**) Ni/La_2_Ce_2_O_7_(CP 500), (**b**) Ni/La_2_Ce_2_O_7_(CP 600), and (**c**) Ni/La_2_Ce_2_O_7_(CP 700); particle size distribution of reduced (**a’**) Ni/La_2_Ce_2_O_7_(CP 500), (**b’**) Ni/La_2_Ce_2_O_7_(CP 600), and (**c’**) Ni/La_2_Ce_2_O_7_(CP 700).

The reduced Ni/La_2_Ce_2_O_7_(CP T) catalysts were further characterized by TEM, and the results are shown in Figure 11. It is seen that there is no significant difference in the morphology of the different supports, which is consistent with XRD results (Figure 8c). In addition, Ni particles are clearly observed on the surface of all samples (as shown by the yellow circles in Figure 11). From the statistical average Ni particle size, the average Ni particle size is in the following order: Ni/La_2_Ce_2_O_7_(CP 700) > Ni/La_2_Ce_2_O_7_(CP 500) > Ni/La_2_Ce_2_O_7_(CP 600), and the Ni particle size distribution on the Ni/La_2_Ce_2_O_7_(CP 600) is more concentrated. This indicates that the active component, Ni, is more effectively dispersed on the La_2_Ce_2_O_7_(CP 600) support. For the Ni/La_2_Ce_2_O_7_(CP 700) catalyst, the average particle size of the active component, Ni, is the largest and the particle distribution is the widest, indicating that the La_2_Ce_2_O_7_(CP 700) support is less favorable for dispersing the active component due to its larger crystal size compared to the other two.

From the above, it can be recognized that the calcination temperature of the support also affects the particle size of La_2_Ce_2_O_7_ support prepared using the co-precipitation method, with the order of Ni/La_2_Ce_2_O_7_(CP 700) > Ni/La_2_Ce_2_O_7_(CP 500) > Ni/La_2_Ce_2_O_7_(CP 600). Moreover, the BET surface area of the support decreases with the increase in particle size. However, an appropriate particle size leads to stronger metal–support interactions and more surface O vacancies.

#### 2.2.2. The Performance of Ni/La_2_Ce_2_O_7_(CP T)

**Figure 12:**
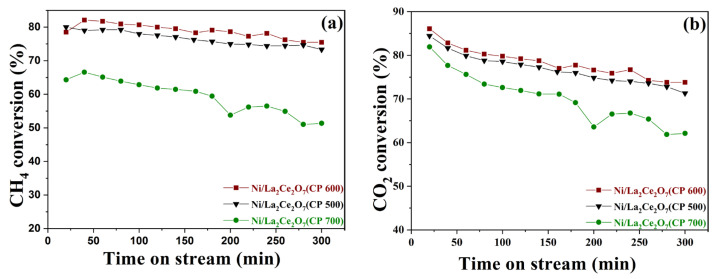
Dependence of catalytic performance of reduced Ni/La_2_Ce_2_O_7_(CP T) catalysts on reaction time. (**a**) CH_4_ conversion; (**b**) CO_2_ conversion. Reaction condition: T = 700 °C, CH_4_/CO_2_ = 1, GHSV = 48,000 mL/(g∙h).

The as-prepared Ni/La_2_Ce_2_O_7_(CP T) catalysts for DRM performance were tested at 700 °C with a ratio of CH_4_/CO_2_ = 1, and the results are shown in Figure 12. It can be seen that the DRM performance of the catalysts has a large difference. The Ni/La_2_Ce_2_O_7_(CP 600) catalyst demonstrates the best activity, while the Ni/La_2_Ce_2_O_7_(CP 700) catalyst exhibits the worst activity. Based on the previous analysis, it seems that the differences in physical properties such as the particle size and specific surface area of the three catalysts are not significant. Therefore, the contrasting performance is mainly attributed to the differences in chemical properties of the catalyst surfaces. H_2_-TPR, XPS and TEM analyses (Figure 9a, Table 4 and Figure 11) revealed that the interaction between the active component, Ni, and the support is more similar in Ni/La_2_Ce_2_O_7_(CP 500) and Ni/La_2_Ce_2_O_7_(CP 600) catalysts. However, La_2_Ce_2_O_7_(CP 600) with more adsorbed oxygen species is more favorable for dispersing the active component, which is beneficial for promoting the cleavage of CH_4_. From the results of CO_2_-TPD and XPS, the Ni/La_2_Ce_2_O_7_(CP 600) catalyst has more strongly basic sites and more surface oxygen vacancies, which are more advantageous for the adsorption and activation of CO_2_. Rightly, the Ni/La_2_Ce_2_O_7_(CP 600) catalyst has superior DRM reaction performance. For the Ni/La_2_Ce_2_O_7_(CP 700) catalyst, the interaction between the active component and the support is weak, resulting in poor dispersion of the active component on the La_2_Ce_2_O_7_(CP 700), which is unfavorable to the CH_4_ cracking; moreover, the surface basicity of this catalyst is weak and the relative content of oxygen vacancies is low, which is not favorable for the adsorption and activation of CO_2_; thus, the DRM reaction performance of Ni/La_2_Ce_2_O_7_(CP 700) catalyst is notably poor. Combined with XRD and N_2_ sorption at low temperatures (Figure 8 and Table 3), it is concluded that the optimal particle size of the pyrochlore support is beneficial for both enhancing Ni dispersion and maintaining a balance between carbon deposition and the removal rate in DRM.

#### 2.2.3. Characterization of the Spent Ni/La_2_Ce_2_O_7_(CP T)

**Figure 13:**
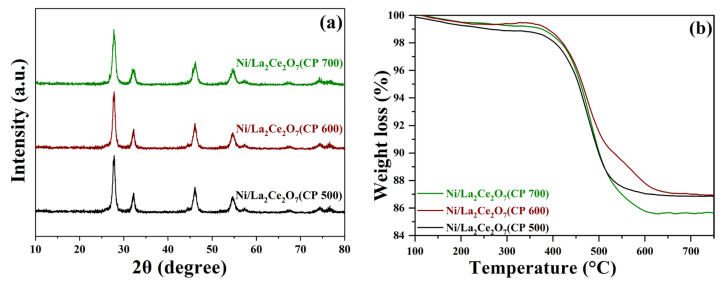
XRD (**a**) and TG profiles (**b**) of spent Ni/La_2_Ce_2_O_7_(CP T) catalysts.

Figure 13 gives the characterization results of the spent Ni/La_2_Ce_2_O_7_(CP T) catalysts. As the XRD patterns in Figure 13a outline, the crystalline structure of the support does not change significantly during the reaction, indicating that the catalysts have good thermal stability. The DTA curves of the spent catalysts (Figure 13b) notably suggest that the Ni/La_2_Ce_2_O_7_(CP 700) with the poorest performance has the largest percentage of carbon weight loss. Moreover, the weight loss ratios of the three catalysts are in the opposite order of their activities. The Ni/La_2_Ce_2_O_7_(CP 600) catalyst presents minimal carbon accumulation, probably ascribed to its relatively high Ni dispersion and more oxygen species on the surface. From the DTA curves (Figure S2), the three catalysts show exothermic peaks at approximately 475 °C, demonstrating that the same type of carbon accumulation exists on the three catalysts, which is assigned to C_β_. The spent Ni/La_2_Ce_2_O_7_(CP 600) shows a slightly lower temperature exothermic peak (Figure S2), indicating that the carbon deposited on this catalyst is more reactive compared to the other two catalysts.

## 3. Materials and Methods

### 3.1. Catalyst Preparation

#### 3.1.1. La_2_Ce_2_O_7_ Supports Preparation

La_2_Ce_2_O_7_ supports were prepared by using glycine nitrate combustion (GNC), sol–gel (S-G) methods and co-precipitation (CP), respectively, and accordingly defined as La_2_Ce_2_O_7_(GNC), La_2_Ce_2_O_7_(S-G), and La_2_Ce_2_O_7_(CP).

For the preparation of La_2_Ce_2_O_7_(GNC), it is described as follows. Simply, the phased Ce(NO_3_)_3_∙6H_2_O and La(NO_3_)_3_∙6H_2_O with a molar ratio of 1:1 were dissolved into deionized water to obtain 0.1 mol/L of Ce^3+^ + La^3+^ aqueous solution. Then, the glycine was added into the above solution at a 1:2 ratio of Ce/glycine, continuously stirring for 30 min until complete dissolution. The resulting solution was slowly placed in the water bath and heated to 80 °C for slow evaporation until forming sticky gel. After that, the gel was transferred to an oven heated to 220 °C for the occurrence of combustion. Subsequently, the obtained fluffy power was further calcined at 500 °C for 4 h, obtaining the La_2_Ce_2_O_7_(GNC) support.

La_2_Ce_2_O_7_(S-G) support was obtained through the following operation. First, Ce(NO_3_)_3_∙6H_2_O and La(NO_3_)_3_∙6H_2_O with a molar ratio of 1:1 were dissolved into deionized water to obtain 0.1 mol/L of Ce^3+^ + La^3+^ aqueous solution, continuously stirring for 30 min; then, the citric acid with 1.2 times the La amount was added into the above solution under stirring. After that, the pH of the solution was adjusted by slowly dropping 37% ammonia aqueous solution to 2. Followingly, the resulting solution was dried at 80 °C until forming sticky gel. After being further dried for 12 h at 130 °C, the gel was calcined at 500 °C for 4 h, thereafter obtaining the La_2_Ce_2_O_7_(S-G) support.

The La_2_Ce_2_O_7_(CP) support was prepared as described in the following process. Typically, Ce(NO_3_)_3_∙6H_2_O and La(NO_3_)_3_∙6H_2_O aqueous solution was obtained as the above operation. Then, the Ce^3+^ and La^3+^ ions were precipitated by adding 37% ammonia aqueous solution dropwise until a pH of 10 along with vigorous stirring. After additional stirring for 30 min, the suspension was filtered and washed several times with deionized water. Subsequently, the resulting cake was dried at 110 °C for 12 h and further calcined at 500 °C for 4 h. Based on different calcination temperatures, the above three supports were defined as La_2_Ce_2_O_7_(CP 500), La_2_Ce_2_O_7_(CP 600) and La_2_Ce_2_O_7_(CP 700).

#### 3.1.2. Preparation of Ni/La_2_Ce_2_O_7_ Catalysts

The present catalysts were all prepared using a wet-impregnation method. Simply, well-weighted Ni(NO_3_)_2_∙6H_2_O was dissolved into 30 mL alcohol with the ultrasonic assistance to impregnate the above-prepared La_2_Ce_2_O_7_ support fine powder. The resulting suspension was stirred at room temperature for 2 h, and the slurry was dried under vacuum (in a rotary evaporator) and further dried at 110 °C overnight. The collected powder was calcined at 500 °C for 4 h. The obtained catalysts were denoted as Ni/La_2_Ce_2_O_7_. Incidentally, the Ni loading of the catalysts was 5wt% unless otherwise specified.

### 3.2. Catalyst Characterization

X-ray diffraction (XRD) patterns of as-prepared samples were recorded using an X-ray diffractometer (Rigaku MiniFlex, Tokyo, Japan) with Cu Kα radiation (40 kV, 15 mA). The Debye–Scherrer equation was used to calculate the crystalline size of the particles.

The textural parameters of the catalysts were obtained on Micromeritics ASAP 2020 (Norcross, GA, USA) through N_2_ sorption at a low temperature (−196 °C). The specific surface area and pore size distribution were calculated, respectively, based on the BET model and the Brunauer–Emmet–Teller (BJH) model using the desorption branch.

Transmission electron microscope (TEM) images of the samples were viewed on JEOL JEM-2100 (Tokyo, Japan) at 200 kV. The sample was first dispersed in ethanol using ultrasound irradiation. Then, the resulting suspension was dropped onto a carbon-coated copper grid (200 mesh) and air dried.

X-ray photoelectron spectroscopy (XPS) measurement was performed on an Shimadzu Axis Ultra Dld instrument (Kyoto, Japan) with Al Kα radiation. The binding energy (BE) was calibrated against the C1s signal (284.8 eV) of contaminant carbon.

The reducibility of the catalysts was characterized using the H_2_-temperature-programmed reduction (H_2_-TPR) technique on a Xianquan chemisorption instrument (TP-5080) (Tianjin, China). A measure of 100 mg of catalyst was pretreated at 300 °C under a flow of Ar (30 mL/min) for 1 h to remove possible humidity and impurities and then cooled to 50 °C. Subsequently, the flow gas was switched to a 10 vol%H_2_/N_2_ flow (30 mL/min). After a flat baseline, the temperature-programmed reduction was carried out from 50 °C to 700 °C with a ramp of 10 °C/min.

CO_2_ temperature programmed desorption (CO_2_-TPD) was examined on a Xianquan chemisorption instrument (TP-5080) (Tianjin, China). Briefly, the sample after removing humidity was pre-reduced at 700 °C for 60 min at 10vol% H_2_/Ar flow then cooled to 50 °C with an Ar atmosphere. After that, the sample was exposed in CO_2_ flow (30 mL/min) for 30 min. Then, pure Ar (30 mL/min) was introduced until the baseline of the TCD was stable. Finally, CO_2_ desorption was carried out at a heating rate of 10 °C/min within a range of 50–700 °C, wherein the CO_2_ desorption signals were monitored using the TCD detector.

Thermogravimetric analysis (TG) and differential thermal analysis (DTA) was used to monitor the weight loss of the spent catalysts with the temperature rising, carried out using a Rigaku TG-DTA 8120 thermogravimetric analyzer (Tokyo, Japan). The sample (10 mg) was heated from room temperature to 850 °C at a rate of 10 °C/min under an air atmosphere.

### 3.3. Catalyst Test

DRM reaction was performed on a quartz fixed-bed microreactor (Φ 8 mm) system, in which a cannula was equipped along the axis to fix thermocouple. The catalyst of 200 mg (20~40 meshes) was diluted with quartz sand (20~40 meshes) of 1000 mg. Before each test, the catalyst was pre-reduced at 700 °C for 60 min under 10% H_2_/Ar (*v*/*v*, 30 mL/min). Subsequently, it was switched to the mixed gas of CH_4_ and CO_2_ (*v*/*v* = 1/1) with GHSV = 48,000 mL/(g∙h) to start the reaction. The effluent gases at the outlet were analyzed online using a gas chromatograph.

The conversions of CH_4_ and CO_2_ and H_2_/CO ratio were calculated using Equations (2)–(4):

(3) $$CH_{4}\;conversion\left(\%\right)=\frac{V_{CH_{4},\;in}-V_{CH_{4},out}}{V_{CH_{4},\;in}}\times 100\%$$

(4) $$CO_{2}\;conversion\left(\%\right)=\frac{V_{CO_{2},in}-V_{CO_{2},out}}{V_{CO_{2},in}}\times 100\%$$

(5) $$H_{2}/COratio=\frac{V_{H_{2},out}}{V_{CO,out}}$$

where V_i,in_ is the volume rate of each fed gas at the inlet, V_i,out_ is the volume rate of each effluent gas at the outlet, and i represents the gas component.

## 4. Conclusions

Pyrochlore-type La_2_Ce_2_O_7_ supports were prepared using glycine nitrate combustion (GNC), sol–gel (S-G) methods and co-precipitation (CP), respectively, and a series of La_2_Ce_2_O_7_(CP) supports were prepared at different calcination temperatures. These supports were used to disperse the Ni component so as to fabricate the catalysts for DRM. The following important results were obtained:

(1) Compared with the catalysts prepared using sol–gel and combustion methods, the precipitated La_2_Ce_2_O_7_(CP 500) catalysts (Ni/La_2_Ce_2_O_7_(CP 500)) show the best DRM reactivity and good stability after Ni loading.
(2) The supports prepared through different methods have different particle sizes and surface properties. Moreover, improving the calcination temperature can promote the aggregation of the support with a small particle size (below 95 nm) but has no influence on the support with a large particle size (such as La_2_Ce_2_O_7_-SG with 229 nm). Pre-reduction at 700 °C also leads to the aggregation of La_2_Ce_2_O_7_ support particles calcined below 700 °C.
(3) An appropriate particle size (68 nm) of the support is beneficial for enhancement of the metal–support interaction and improvement of Ni dispersion, effectively promoting the cracking of CH_4_. Meanwhile, supports with an appropriate particle size have more oxygen vacancies, leading to the presence of more surface-adsorbed oxygen species and basic sites for CO_2_ adsorption, accelerating carbon removal on the catalyst surface. Thus, the catalyst obtained with La_2_Ce_2_O_7_(CP 600), which has the most appropriate size, shows optimal catalytic performance in DRM among the representative catalysts.

## Figures and Tables

**Table 1:** The physical properties of the La_2_Ce_2_O_7_ supports and supported catalysts.

Sample	Specific Surface Area (m^2^/g)	Pore Volume (cm^3^/g)	Average Pore Diameter (nm)	Crystalline Size ^a^ (nm)
La_2_Ce_2_O_7_(CP 500)	35.2	0.1105	12.6	56
La_2_Ce_2_O_7_(GNC)	20.5	0.0575	11.2	60
La_2_Ce_2_O_7_(S-G)	12.9	0.0398	12.3	229
Ni/La_2_Ce_2_O_7_(CP 500)	33.5	0.1127	13.4	95 *
Ni/La_2_Ce_2_O_7_(GNC)	16.7	0.0613	14.7	108 *
Ni/La_2_Ce_2_O_7_(S-G)	13.8	0.0549	15.9	224 *

^a^ Calculated using the Scherrer equation according to the (111) crystal surface reflection of XRD results. * The crystalline size of the reduced catalysts.

**Table 2:** The XPS quantification results of the Ni/La_2_Ce_2_O_7_ catalysts.

Catalyst	Ce^3+^ (%)	Ni Atomic Concentration (%)	O 1s B.E (eV)/Relative Amount (%)				(α + β)/γ
			O_2_^−^ (α)	CO_3_^2−^	O_2_^2−^ (β)	O^2−^ (γ)	
Ni/La_2_Ce_2_O_7_(S-G)	21	4.06	533.0/2.9	531.4/50.3	529.5/7.0	528.7/39.8	0.25
Ni/La_2_Ce_2_O_7_(GNC)	25	4.66	533.0/3.6	531.3/55.1	529.4/7.2	528.6/34.1	0.32
Ni/La_2_Ce_2_O_7_(CP 500)	27	4.69	533.0/7.8	531.4/40.2	530.0/13.4	528.7/38.6	0.54

**Table 3:** The physical properties of the La_2_Ce_2_O_7_ supports and supported catalysts.

Sample	Specific Surface Area (m^2^/g)	Pore Volume (cm^3^/g)	Average Pore Diameter (nm)	Crystalline Size ^a^ (nm)
La_2_Ce_2_O_7_(CP 500)	35.21	0.1105	12.55	56
La_2_Ce_2_O_7_(CP 600)	27.91	0.1142	16.36	68
La_2_Ce_2_O_7_(CP 700)	26.21	0.1065	16.26	88
Ni/La_2_Ce_2_O_7_(CP 500)	33.53	0.1127	13.44	95 *
Ni/La_2_Ce_2_O_7_(CP 600)	28.00	0.1156	16.51	87 *
Ni/La_2_Ce_2_O_7_(CP 700)	28.24	0.1205	17.06	91 *

^a^ Calculated using the Scherrer equation according to the (111) crystal surface reflection of XRD results. * The crystalline size of the reduced catalysts.

**Table 4:** Quantities of surface oxygen species based on XPS.

Catalyst	O 1s B.E (eV)/Relative Amount (%)				(O_2_^−^ + O_2_^2−^)/O^2−^
	O_2_^−^	CO_3_^2−^	O_2_^2−^	O^2−^	
Ni/La_2_Ce_2_O_7_(CP 500)	533.0/7.80	531.4/40.16	530.0/13.39	528.7/38.64	0.54
Ni/La_2_Ce_2_O_7_(CP 600)	533.0/8.63	531.3/43.50	529.6/12.16	528.6/35.7	0.58
Ni/La_2_Ce_2_O_7_(CP 700)	533.1/2.89	531.3/52.43	529.4/7.55	528.6/37.12	0.28

## Data Availability

The original contributions presented in the study are included in the article and Supplementary Materials, further inquiries can be directed to the corresponding author.

## Supplementary Materials

The following supporting information can be downloaded at: https://www.mdpi.com/article/10.3390/molecules29081871/s1, Figure S1: N_2_ isotherms and pore distribution of the prepared supports and Ni-loaded catalysts. Figure S2: DTA profiles of spent Ni/La_2_Ce_2_O_7_(CP T) catalysts.
