# Supplementary material for: Dry Reforming of Methane over Pyrochlore-Type La2Ce2O7-Supported Ni Catalyst: Effect of Particle Size of Support
Source: Molecules. 2024 Apr 19;29(8):1871. doi: 10.3390/molecules29081871 (PMC11054847; doi:10.3390/molecules29081871)
Supplement: Supplementary file 1 [file molecules-29-01871-s001.zip › molecules-2947371-supplementary.pdf]

Supporting information

Dry reforming of methane over pyrochlore-type $La_2Ce_2O_7$ supported Ni catalyst: effect of particle size of support

Zeling Zhou[a,b,&], Chao Li[a,b,&], Junfeng Zhang[a,*], Qiliang Gao[a,b], Jiahao Wang[a,b], Qingde Zhang[a], Yizhuo Han[a,*]

[a] State Key Laboratory of Coal Conversion, Institute of Coal Chemistry, Chinese Academy of Sciences, Taiyuan, 030001, China

[b] University of Chinese Academy of Sciences, Beijing, 100049, China

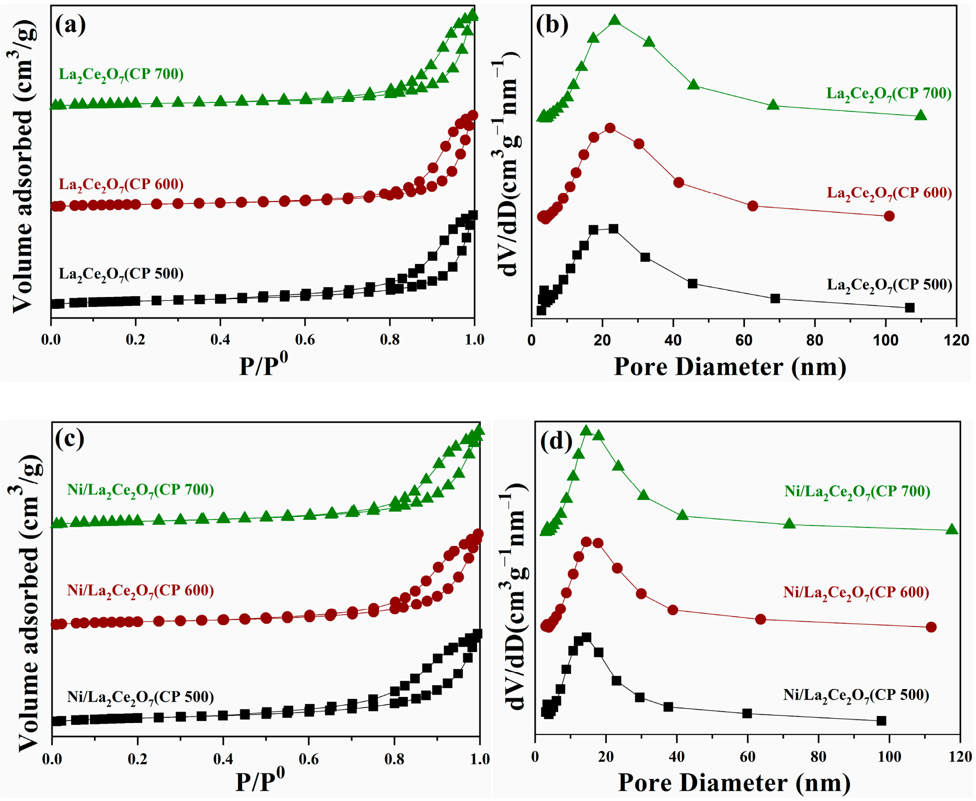

Figure S1 N_2 isotherms and pore distribution of the prepared supports (a and b) and Ni-loaded catalysts (c and d).

*: Corresponding authors. &:These authors contributed equally to this work

Institute of Coal Chemistry, Chinese Academy of Sciences, Taiyuan, 030001, China. Tel: +86-351-4049747, E-mail: hanyz@sxicc.ac.cn (Y.Z. Han); zhangjf@sxicc.ac.cn (J.F. Zhang)

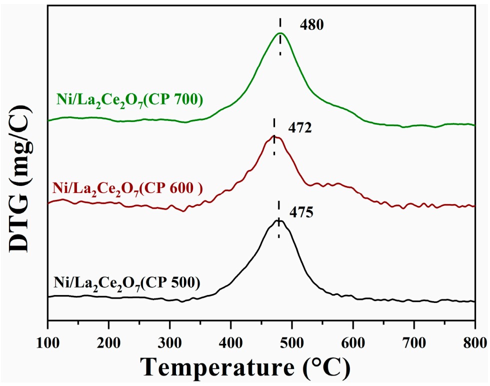

Figure S2 DTA profiles of spent $Ni/La_2Ce_2O_7$(CP T) catalysts
